# The Role of HPV and Hormone in Cervical Precancer and Cancer: Molecular Pathophysiology and Cell Biology of Disease and Treatment

**DOI:** 10.32604/or.2026.078219

**Published:** 2026-05-21

**Authors:** Pei-Yu Kao, Jie-Hong Chen, Kuo-Hu Chen

**Affiliations:** 1 Department of Obstetrics and Gynecology, Taipei Tzu-Chi Hospital, The Buddhist Tzu-Chi Medical Foundation, New Taipei City, Taiwan; 2 School of Medicine, College of Medicine, MacKay Medical University, New Taipei City, Taiwan; 3 School of Medicine, Tzu-Chi University, Hualien, Taiwan

**Keywords:** Cervical cancer, cervical precancer, human papillomavirus, estrogen signaling, tumor microenvironment

## Abstract

Cervical cancer remains a major global health challenge despite advances in human papillomavirus (HPV) vaccination, screening, and treatment. Persistent infection with high-risk HPV types, particularly HPV16 and HPV18, is a necessary cause of cervical cancer; however, only a small fraction of infections progress to malignancy, indicating the importance of additional cofactors. Increasing evidence identifies estrogen signaling as a critical modifier of HPV-driven carcinogenesis. Estrogen acts synergistically with HPV oncogenes E6 and E7 to promote genomic instability, immune evasion, and tumor progression, largely through effects on the tumor microenvironment (TME). This review aims to clarify and summarize current knowledge on the interplay between HPV biology, estrogen signaling, and the cervical cancer microenvironment, discussing HPV structure, life cycle, and mechanisms of oncogenesis including viral genome integration, disruption of tumor suppressor pathways, and induction of chromosomal instability. Particular emphasis is placed on estrogen receptor signaling, highlighting the shift from tumor cell–intrinsic estrogen receptor α (ERα) expression to a paracrine, stromal-driven mechanism during disease progression. Estrogen signaling in cancer-associated fibroblasts, myeloid-derived suppressor cells, and regulatory T cells fosters an immunosuppressive microenvironment that supports viral persistence and malignant transformation. Both classical genomic and rapid non-genomic estrogen pathways are discussed, as well as their roles in immune modulation and DNA damage responses. Furthermore, there is emerging evidence linking estrogen signaling to HPV-induced genomic instability through pathways involving G protein-coupled estrogen receptor 1 (GPER1), estrogen receptor α36 (ERα36), High Mobility Group AT-Hook 2 (HMGA2), and wings apart-like (WAPL). Finally, the review contextualizes these molecular insights within contemporary clinical management, summarizing standard treatments and recent advances in targeted and immunotherapies, including bevacizumab and pembrolizumab. Understanding estrogen-driven stromal–immune crosstalk in HPV-associated cervical carcinogenesis may uncover novel therapeutic opportunities. Integrating hormonal modulation with immunotherapy and other targeted strategies represents a promising avenue to improve outcomes, particularly in advanced and treatment-resistant disease.

## 1 Introduction

Globally, cervical cancer continues to be a public health burden despite decades of progress in screening, vaccination, and treatment. In 2020, an estimated 604,127 women were diagnosed with cervical cancer, and 341,831 died from the disease. A clear socioeconomic gradient is evident, with countries of low Human Development Index (HDI) exhibiting threefold higher incidence and sixfold higher mortality compared with very high HDI countries. Although the incidence of cervical cancer has declined and stabilized at low levels in many high-income regions since the mid-2000s, several countries in eastern Africa and eastern Europe continue to experience rising trends [1,2]. These data show the urgent need to accelerate progress toward the WHO elimination target of fewer than four cases per 100,000 women-years [1,2].

Cervical cancer is related to persistent infection by high-risk human papillomaviruses (HR-HPVs), particularly HPV16 and HPV18. These are recognized as the essential cause of cervical precancer and cancer [3]. Although HPV infection is common, most infections are transient and cleared by host immunity [4,5]. Only a minority progress to high-grade cervical intraepithelial neoplasia (CIN) or invasive carcinoma. This may imply that additional cofactors are required for malignant transformation.

Accumulating evidence identifies estrogen signaling as a critical cofactor in cervical carcinogenesis, functioning synergistically with the viral oncoproteins E6 and E7 to drive genomic instability, promote epithelial transformation, and reshape the tumor microenvironment (TME) [6,7,8]. As cervical lesions advance, surrounding stromal cells such as cancer-associated fibroblasts (CAFs), myeloid-derived suppressor cells (MDSCs), and regulatory T cells (Tregs) express estrogen receptor α (ERα). Estrogen signaling then accelerates carcinogenesis. These microenvironmental changes support viral persistence, tumor progression, and resistance to host immune clearance [9,10].

From a therapeutic perspective, management of cervical cancer traditionally relies on surgical treatment for early-stage disease and concurrent chemoradiation therapy for locally advanced stages [11,12]. In recent years, advances in targeted and immunologic therapies have significantly expanded treatment options, particularly for recurrent, persistent, or metastatic disease. Bevacizumab has demonstrated survival benefits when it is added to standard chemotherapy [13,14]. Likewise, pembrolizumab has emerged as an important therapeutic agent, which is approved both in combination with chemoradiation for locally advanced disease and for PD-L1–positive recurrent or metastatic tumors [15]. These developments highlight the growing role of molecularly guided and immunotherapeutic strategies in cervical cancer treatment.

This review aims to clarify the underlying mechanisms of cervical precancer and cancer, including the role of estrogen signaling in modulating HPV-driven cervical carcinogenesis, with emphasis on stromal–immune crosstalk and implications for targeted therapy.

## 2 Human Papillomavirus (HPV): Structure and Action

Human papillomaviruses (HPVs) are small, double stranded DNA viruses. There are numerous types of HPV. The existing HPVs are categorized into 5 different genotypes: α, β, γ, μ, and ν groups [16]. Each genotype differs from another by less than 90% sequence similarity in L1 gene region [17,18,19]. Among these HPVs, the so-called high-risk human papillomaviruses (HR-HPVs) have been classified as oncogenic. HPV16 and HPV18 are both HR-HPVs categorized in the α group. HPV16 accounts for approximately 60% of cervical cancers and is more related to squamous-cell carcinomas. On the other hand, HPV18 causes approximately 15% of cases and is more related to adenocarcinoma. The rest of cervical cancers are caused by the other HR-HPVs [20,21].

All HPVs contain a circular DNA genome of ~8 kb in size, encoding around eight open-reading frames (ORFs) [22]. These ORFs can be divided into three distinct regions: the upstream regulatory region (URR), the early (E), and the late (L) gene regions. The URR region is also called long control region (LCR), which does not encode any protein. 

**Figure 1:**
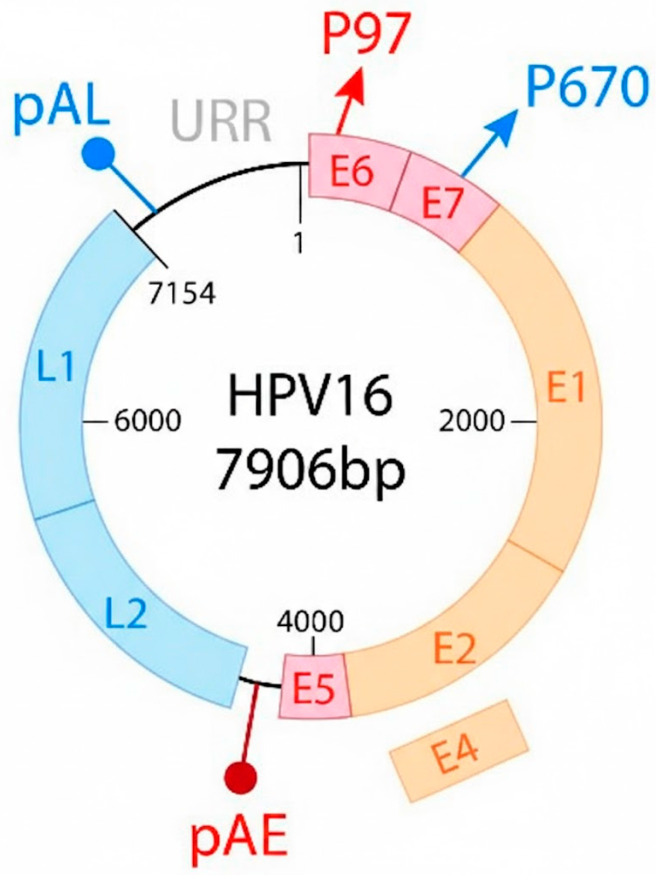
The circular genome (7906 bp) of human papillomavirus (HPV) 16 enclosed within a simplified viral capsid structure, showing the early gene region encoding E1, E2, E4, E5, E6, E7 proteins, the late gene region encoding two (L1 and L2) viral structural proteins, early (pAE) and late (pAL) polyadenylation sites. E6, E7, E5 (red boxes): early oncogenes; E1, E2 (orange boxes): replication and transcription regulation functions; L1, L2 (blue boxes) encoding capsid proteins; URR (grey): Upstream Regulatory Region; P97 promoter (red arrow): driving early gene expression; pAE (red circle on a line): early polyadenylation; P670 promoter (blue arrow): driving late gene expression; pAL (blue circle on a line): late polyadenylation.

Fig. 1 illustrates the circular genome of HPV 16. The early gene region encodes E1, E2, E1^E4, E5, E6, E7, and E8^E2 proteins, which are responsible for infection, viral replication, and oncogenesis [23]. The late gene region encodes two viral structural proteins, L1 and L2 proteins, that are responsible for the structural components. The protein shell is made of 72 pentamers, with each pentamer composed of five L1 proteins and a small number of L2 proteins. The early gene regions E6 and E7 are responsible for inactivation of the tumor supressor gene p53 (Table 1). In addition, the HPV genome contains early (PE) and late (PL) promoters, as well as early (pAE) and late (pAL) polyadenylation sites. 

**Table 1:** The Structures and Functions of human papillomavirus (HPV) 16.

ORF	Function
**E1**	Origin binding protein. ATP-dependent helicase that binds the viral origin to unwind DNA and initiate replication.
**E2**	Assists E1 in replication initiation; represses E6/E7 expression via binding to LCR and early promoters
**E4**	Mainly expressed as the E1^E4 fusion protein; disrupts cytokeratin structure to support viral assembly and release. Functions primarily in the late phase of the viral life cycle
**E5**	Small hydrophobic membrane protein that promotes cell proliferation, immune evasion, and tumor progression by enhancing EGFR signaling
**E6**	Promotes oncogenesis by degrading p53, activating multiple proliferative and anti-apoptotic pathways; facilitates immune evasion and cell immortalization through hTERT activation.
**E7**	Promotes oncogenesis by degrading pRb, disrupting cell cycle control, enhancing proliferation, invasion, immune evasion, and EMT; it also cooperates with E6 to alter metabolism and support cell immortalization.
**E8**	Forms the E8^E2 fusion protein; inhibits viral transcription and replication to regulate the viral life cycle.
**L1**	Major structural protein; forms the viral capsid used in prophylactic vaccines.
**L2**	Minor capsid protein; assists with viral genome packaging, entry, and intracellular transport.

Note: ORF: open reading frame; ATP: Adenosine Tri-phosphate; LCR: long control region; EGFR: Epidermal Growth Factor Receptor; hTERT: Human Telomerase Reverse Transcriptase; EMT: Epithelial−Mesenchymal Transition.

**Figure 2:**
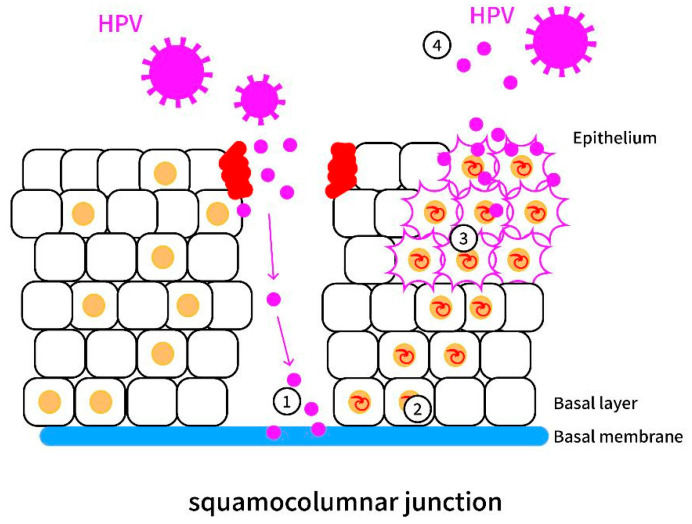
The mechanism of human papillomavirus (HPV) infection and the life cycle of HPV, staging from (1) initial infection, (2) genome maintenance, and (3) vegetative amplification to (4) virion assembly and release.

Persistent HR-HPV infection is a significant cause of cervical cancer. The infection begins when microabrasions in the squamocolumnar junction of the cervix allow viral entry into basal epithelia [24,25]. After endocytic entry, the viral genome, escorted by the L2 protein, traffics to the nucleus during mitosis and establishes a low-copy episomal state [26]. The viral life cycle includes four stages: initial infection, genome maintenance in basal cells, vegetative amplification in differentiated layers, and final virion assembly and release (Fig. 2). HPV hijacks host DNA and further results in DNA damage response (DDR) pathways—particularly ATM (ataxia–telangiectasia mutated) and ATR (ataxia-telangiectasia and Rad3-related) signaling—to facilitate replication, especially at fragile chromosomal sites prone to stress [27,28]. 

The receptors for HPV to enter the host cells can be categorized into primary attachment factors and secondary uptake receptors. The molecules of primary attachment factors (the gateway) facilitate the initial tethering of the virus to the host surface or the extracellular matrix (ECM). They include heparan sulfate proteoglycans (HSPGs) and laminin-332. HPV L1 protein binds to the glycosaminoglycan (GAG) chains of HSPGs as the canonical initial entry point. Syndecan-1 is the most highly expressed HSPG in basal keratinocytes. It acts as a primary attachment site and is upregulated during wound healing [29,30]. For another HSPG Glypicans, reports have shown the clinical association of gene polymorphisms with an increased risk of progression from CIN to invasive cancer [30,31]. Laminin-332 is a key component of the basement membrane. *In vivo*, HPV often binds first to laminin-332 exposed via microtrauma before being “handed off” to the keratinocyte [29,32].

Once bound to HSPGs, the virus undergoes conformational changes (mediated by cyclophilin B and furin cleavage of HPV L2) to engage these secondary uptake receptors (the internalizers), which trigger endocytosis. There are three types of uptake receptors including *α*6-Integrins (ITGA6), growth factor receptors (GFRs), and annexin A2 heterotetramer (A2t). As the main secondary uptake receptor, *α*6-Integrins (ITGA6) initiates the intracellular signaling events required for the virus to be internalized via clathrin−dependent or independent pathways [30,33]. Also, HPV can utilize GFRs. The virus-HSPG complex can transactivate these growth factor receptors to induce the Src kinase signaling required for viral entry [34,35]. Furthermore, A2t is a recently identified HPV L2-specific receptor. The interaction between the viral HPV L2 N-terminus and annexin A2 heterotetramer facilitates the trafficking of the viral genome through the endolysosomal system [35,36].

High-risk human papillomaviruses (HPVs) exploit the host’s DDR to facilitate viral genome amplification, particularly during the differentiation-dependent productive phase [37,38]. The viral oncoprotein E7 induces replication stress and genomic instability by inactivating the retinoblastoma protein (pRb), leading to aberrant S-phase reentry and nucleotide depletion [39,40]. This stress activates the ATR (ataxia telangiectasia and Rad3-related) pathway, which responds to stalled replication forks and single-stranded DNA [38,41].

The ATR pathway is a critical sensor of replication stress. In cervical cancer, HPV E7-induced nucleotide depletion causes stalled replication forks and single-stranded DNA gaps. ATR detects these via RPA-coated templates, activating Chk1 to stabilize forks and facilitate the high-fidelity amplification of viral episomes at the expense of host genomic stability. During this pathway, Rad3 is an essential PI3K-related kinase (PIKK) homologous to human ATR, functioning as a master regulator of DNA integrity checkpoints. It detects DNA damage (via Rad1/9/17/26/Hus1) and associated phosphorylation activates downstream kinases (Chk1/Cds1) to arrest the cell cycle, ensuring repair before mitosis [38,41]. 

Simultaneously, the ATM (ataxia–telangiectasia mutated) pathway is activated, primarily triggered by the formation of double-strand breaks (DSBs) during uncoordinated viral replication [37,42]. HPV hijacks these signaling cascades by selectively recruiting essential DDR factors—such as the MRN complex, BRCA1, and RAD51—to viral replication centers [38,39]. By sequestering these proteins away from host fragile chromosomal sites, HPV promotes its own high-fidelity replication while simultaneously increasing the host’s susceptibility to chromosomal rearrangements and viral integration [40,42].

In high-risk types such as HPV-16 and HPV-18, viral genome integration into host DNA is a critical event in the oncogenesis process. Integration often disrupts the E2 gene, which cancels the suppression of E6 and E7 oncogenes [43]. The early gene regions E6 and E7 act as oncogenes that promote tumor growth and mailgnant transformation, because their end products (such as E6 proteins) can bind to two host cell proteins including p53 and E6-associated proteins (E6AP) to inhibit the function of p53. E6 promotes degradation of the tumor suppressor p53 via the E6AP ubiquitin ligase, impairing DNA damage response and apoptosis [44,45]. Synergistically, E7 binds and inactivates the retinoblastoma protein (pRb), releasing E2F transcription factors and driving uncontrolled entry into S phase [46].

Additionally, integration can generate fusion transcripts, induce epigenetic dysregulation, and form extrachromosomal DNA (ecDNA) that amplifies oncogene expression [26]. Moreover, HPV evades immune detection by downregulating major histocompatibility complex (MHC) class I, and recruiting immunosuppressive regulatory T cells [47,48]. Together, these mechanisms contribute to viral persistence, immune evasion, and the multistep process of cervical carcinogenesis.

## 3 The Concentration of Estrogen in Tumor Microenvironment (TME)

The concentration of estradiol (E2) within cervical tissues may significantly influence the tumor microenvironment (TME) and carcinogenic processes. While the systemic E2 level may be within normal range, the local concentration of E2 in TME may be higher due to increased production of local estrogen. Aromatase is the key enzyme that catalyzes the transformation of androstenedione into estrone (E1) and testosterone into E2 [49,50]. In the local production of E2, steroid sulfatase (STS), and 17β-hydroxysteroid dehydrogenase (17β-HSD) also play an important part. A study found that STS activity was much higher *in vitro* in the HPV-positive cervical carcinoma cell line Hela, leading to the transformation of E1-sulphate to E1. 17β-HSD then transforms E1 to E2 [51]. Elevated intratumoral E2 levels may potentiate estrogen receptor signaling even in the absence of systemic hyperestrogenism, thereby promoting cervical carcinogenesis. 

## 4 Estrogen Signaling and Estrogen-Induced Immuno Response

### 4.1 Estrogen Signaling

Estrogen signals through both classical genomic and non-genomic pathways. In the genomic pathway, the classical ER is involved. The classical ER is divided into two subtypes, ERα and ERβ, which are encoded by Estrogen Receptor 1 (ESR1) and Estrogen Receptor 2 (ESR2) genes, respectively. ERα and ERβ mainly express in plasma membrane and nucleus, and both of them contain a DNA-binding domain. ERα expression may increase at early stages of cancer, and acts as a tumor promoter [52]. In the classical genomic pathway, estrogen enters the cell and binds to intracellular ERα or ERβ, forming hormone-receptor complexes. The complexes translocate to the nucleus and bind to estrogen-responsive elements (EREs), activating gene transcription [53].

In the non-genomic pathway, estrogen binds to membrane-bound receptors such as G protein-coupled estrogen receptor 1 (GPER1), also known as G protein-coupled receptor 30 (GPR30) [54]. Responsible for context-dependent signal transactivation. GPER1 (GPR30) is a G protein-coupled receptor that provides an alternative pathway for estrogenic action independent of classical nuclear receptors [55,56]. In cervical cancer, GPER1 activation leads to the mobilization of intracellular calcium and the activation of MMP-2/9, which facilitates the release of heparin-binding EGF-like growth factor (HB-EGF) [56]. This results in the transactivation of the EGFR pathway [56,57]. Therefore, the estrogen-GPER1 connection rapidly activates signaling pathways involving cAMP, intracellular calcium, and tyrosine kinase Src without DNA interaction. Eventually, stimulation of GPER-1 results in cell proliferation, survival and invasion [58]. On the other hand, high-risk HPV E6 protein can upregulate GPER1 expression. Once activated, GPER1 modulates the YAP/TAZ transcriptional co-activators of the Hippo pathway [57]. This interaction enhances the Epithelial−Mesenchymal Transition (EMT), increasing the metastatic potential of HPV−transformed cells [56,57].

**Figure 3:**
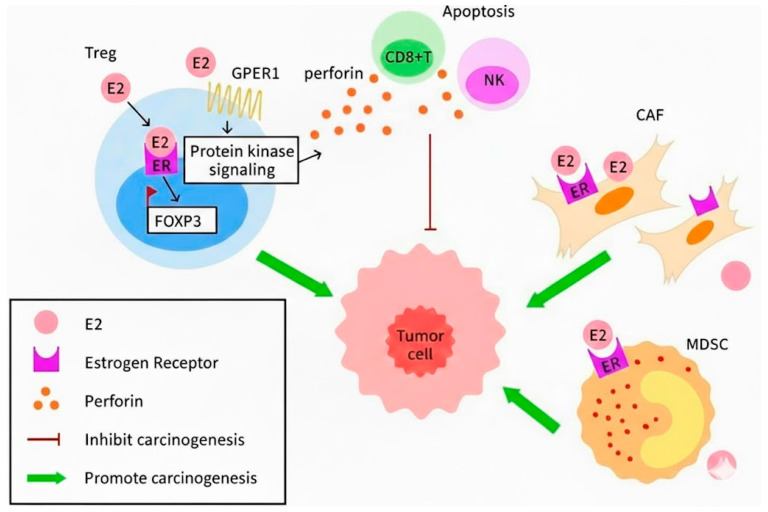
Estrogen signaling in HPV-related cervical precancer and cancer. The diagram illustrates classical genomic and non-genomic estrogen signaling pathways that modulate tumor proliferation, immune suppression (via Tregs and MDSCs), and stromal responses (via CAFs) in the HPV-infected cervical microenvironment. Treg: regulatory T cells; GPER1: G protein-coupled estrogen receptor 1; FOXP3: Forkhead box protein P3; CAFs: cancer-associated fibroblasts; MDSCs: myeloid-derived suppressor cells; CD8^+^: cluster of differentiation 8+; NK: natural killer; E2: estradiol; ER: estrogen receptor.

During the progression of pre-invasive cervical lesions and cervical cancer, estrogen receptor α (ERα) expression is frequently lost in tumor cells [59]. However, ERα expression is retained in the surrounding stromal compartment. Immunohistochemical studies have shown that ERα is expressed in 30–50% of stromal cells, including cancer-associated fibroblasts (CAFs), myeloid-derived suppressor cells (MDSCs), and certain lymphocytes, but is largely absent in tumor cells themselves [59,60]. This implies that estrogen acts in a paracrine mechanism, wherein estrogen influences tumor cells indirectly through stromal cells [10]. Fig. 3 illustrates the classical genomic and non-genomic estrogen signaling pathways in inducing HPV-related cervical precancer and cancer. Activation of ERα in CAFs leads to the secretion of soluble pro-tumorigenic factors, which promote malignant cell proliferation, angiogenesis, epithelial–mesenchymal transition (EMT), and chronic inflammation within the cervical tumor microenvironment.

Cancer-associated fibroblasts (CAFs) constitute a major stromal component of the cervical cancer microenvironment and actively participate in estrogen-driven paracrine signaling. Accumulating evidence indicates that CAFs are not merely structural elements but function as dynamic regulators of tumor progression through secretion of cytokines, chemokines, and extracellular matrix–associated factors that remodel the tumor microenvironment [6,61]. Correlative transcriptomic analyses further suggest that stromal ERα activity is associated with altered expression of chemokines and inflammatory mediators, including CXCL12, CXCL14, and IL-8, implicating CAF-derived signals in stromal–epithelial crosstalk and immune modulation [10].

A distinct subset of periostin-expressing CAFs has been identified in cervical squamous cell carcinoma, correlated with lymph node metastasis and poor clinical outcomes. Periostin-expressing CAFs impair lymphatic endothelial barrier integrity through activation of integrin–FAK/Src signaling and subsequent disruption of VE-cadherin–mediated cell–cell junctions, thereby facilitating lymphatic dissemination [62]. Collectively, these findings support a model in which estrogen-responsive CAFs contribute to tumor progression through paracrine remodeling of the cervical tumor microenvironment, acting in concert with other immunosuppressive stromal components. 

A study in transgenic mouse models has confirmed that the deletion of ERα in stromal cells induced complete regression of cervical dysplasia. This finding underscores the critical contribution of stromal ERα in mediating the tumor-promoting effects of estrogen [63]. Moreover, pharmacologic inhibition of stromal ERα using SERMs (e.g., MPP) or SERDs (e.g., fulvestrant) has been shown to downregulate genes involved in metabolism and cell cycle progression, leading to reduced tumor progression and vascularization [6]. These findings underscore stromal ERα as a critical mediator of estrogen-driven carcinogenesis and suggest its blockers may represent a promising therapeutic target in ERα-negative cervical tumors.

### 4.2 Estrogen-Induced Immuno Response

Estrogen also plays a role in immune response in cervical carcinogenesis by directly acting on myeloid-derived suppressor cells (MDSCs) and regulatory T cells (Treg). These are two important infiltrating cells related to immunosuppression. Estrogens induce deregulated myelopoiesis and also enhance the mobility of MDSCs and their inherent immunosuppressive character [64]. MDSCs are accumulated during tumor progression, thus forming an immunosuppressive tumor microenvironment (TME) [65,66]. Mechanistically, there are multiple downstream pathways for MDSC to induce immunosuppressive effects. MDSCs produce arginase-1 (Arg-1), which causes depletion of L-arginine, and results in downregulation of the CD247 expression and impaired T-cell receptor signaling. MDSCs also express high levels of inducible nitric oxide synthase (iNOS), which produces nitric oxide that nitrates T-cell receptors and disrupts antigen-specific recognition. Increased reactive oxygen species (ROS) generation further compromises T-cell viability and effector function. Collectively, these mechanisms lead to profound functional suppression of cytotoxic T cells rather than simple immune cell depletion [67]. Estrogen has been shown to promote tumor progression through immune-mediated mechanisms independent of tumor cell–intrinsic ERα expression. Kozasa et al. found that in ERα-negative cervical cancer models, 17β-estradiol (E2) stimulates the expansion and mobilization of MDSCs from the bone marrow and enhances their immunosuppressive activity via ERα expressed on stromal and myeloid compartments [9]. Together, these findings support a stromal ERα–MDSC paracrine axis as a critical mediator of estrogen-induced immunosuppression.

Likewise, Tregs (or suppressor T cells) constitute a key immunosuppressive population within the cervical cancer microenvironment. Estrogen signaling has been shown to modulate Treg activity via classical genomic as well as non-genomic pathways, thereby reinforcing immune suppression during cervical carcinogenesis. In the classical genomic pathway, tumor-infiltrating Tregs express ERα and are exposed to elevated local estrogen signaling within the TME [68]. Estrogen binding promotes the formation of E2:ERα complexes, which interact with regulatory elements within the FOXP3 promoter and directly modulate FOXP3 transcription [68]. FOXP3 is a lineage-specific transcription factor that governs Treg development, differentiation, maintenance and function [69]. To date, eight putative estrogen response elements (ERE) within the FOXP3 promoter have been identified, supporting a direct transcriptional role for ERα signaling in Treg regulation. Estrogen-conditioned Tregs increase the secretion of inhibitory cytokines such as interleukin-10 (IL-10) and transforming growth factor-β (TGFβ), thereby suppress effector CD8^+^ T-cell proliferation and cytokine production [68]. These effects allow tumor cells to evade immune surveillance despite the presence of tumor antigens and cytolytic immune components.

In addition to genomic signaling, non-genomic estrogen signaling has been implicated in Treg activation through membrane-associated estrogen receptors, including GPER1 and membrane-bound ERα, leading to activation of intracellular pathways such as PI3K/Akt. The expression of perforin is also found. Treg cells derived from the tumor microenvironment may utilize perforin to traffic granzymes B into the cytosol of natural killer cells and CD8^+^ T cell, and subsequently induce apoptosis of NK cells and CD8^+^ T cell. Working as destructors, granzyme B and perforin are therefore relevant for Treg cell-mediated suppression of tumor clearance [70,71].

Estrogen-driven expansion of MDSCs and Tregs may attenuate cytotoxic T-cell activation, following PD-1/PD-L1 blockade, thereby contributing to heterogeneous responses to immunotherapy in cervical cancer.

## 5 HPV-Induced Genomic Instability and the Role of E2 Signaling

Infection with high-risk HPV types is necessary but not sufficient for progression to cervical cancer. Mutations in cellular genes and chromosomal rearrangements induced by genomic instabilities are important contributing events [38]. A genomic analysis of 79 primary squamous cell carcinomas of cervix identified novel somatic mutations in genes such as MAPK1, HLA-B, TP53, and ERBB2. Notably, cervical squamous cell carcinomas had higher frequencies of APOBEC signature mutations than adenocarcinomas [72]. The APOBECs, a group of cytidine deaminases, represent an unusual protein family that can insert mutations in DNA and RNA as a result of their ability to deaminate cytidine to uridine, which may contribute to the onset of cervical cancer [73]. Gene expression levels at HPV integration sites were significantly higher in tumors with HPV integration compared with expression of the same genes in tumors without viral integration at the same site.

Building upon these findings, The Cancer Genome Atlas (TCGA) conducted a large comprehensive genomic study of 228 primary cervical cancers in 2017. Integration of HPV was observed in all HPV18-related samples and 76% of HPV16-related samples, and was associated with structural aberrations and increased target-gene expression. Tumors with high copy number alterations, mostly cervical squamous cell carcinomas, was found to involve 11q22 (YAP1, BIRC2, BIRC3) and 7p11.2 (EGFR) amplification. Tumors with low copy number alterations, mostly adenocarcinomas, was enriched with deletions in TGFBR2 and SMAD4, and gains in ERBB2 (HER2) and KLF5. Samples in both groups showed PD-L1 (CD274) and PD-L2 (PDCD1LG2) amplifications, which significantly correlated with expression of cytolytic markers granzyme A and perforin, suggesting potential responsiveness to immune checkpoint blockade [74].

Further studies have explored how E2 signaling affects genomic instability in cervical carcinogenesis. Ogawa et al. (2023) reported that E2 increases the proliferation of normal endocervical columnar and adenocarcinoma cells via GPER1 (the non-genomic pathway). Then, it leads to increased accumulation of DNA double-strand breaks (DSBs) in high-risk HPV-E6-expressing cells. The increase in DSBs resulted from the impairment of Rad 51 and accumulation of topoisomerase-2-DNA complexes under HPV-E6 expression. In addition, chromosomal aberrations increased in cells with E2-induced DSB accumulation [7].

In the transition from HPV-induced cervical precancer (CIN) to invasive carcinoma, the crosstalk between estrogen signaling and high-risk HPV E6/E7 oncogenes is not merely additive but synergistic [75,76]. The following is an in-depth elaboration on the specific regulatory mechanisms of ERα36, WAPL, and HMGA2 in this oncogenic axis.

ERα36 is a newly identified isoform of ERα, serving as the non-genomic driver of E6/E7 expression. While the classical estrogen receptor (ERα66) often acts as a tumor suppressor or is downregulated in cervical cancer, its 36-kDa variant, ERα36, is frequently overexpressed. ERα36 lacks the intrinsic transcriptional activation domains (AF-1 and AF-2) of the full-length receptor. Instead, it localizes primarily to the plasma membrane and cytoplasm, where it couples with G-proteins to trigger rapid MAPK/ERK and PI3K/Akt signaling [8,77]. In its crosstalk with HPV, ERα36 mediates a positive feedback loop. Estrogen binding to ERα36 induces the transcription of HPV E6 and E7 [77]. Conversely, E7 has been shown to stabilize ERα36 protein levels, creating a self-sustaining oncogenic circuit that promotes cell proliferation and inhibits apoptosis even under low-estrogen conditions [8,77]. A study showed that the overexpression of ERα36 promotes the proliferation, invasion and metastasis of cervical cancer cells induced by E2.

Acting as a chromatin remodeler and “molecular switch”, High Mobility Group AT-Hook 2 (HMGA2) is a downstream target of ERα36 [8]. HMGA2 is a non-histone architectural transcription factor that is nearly undetectable in normal adult tissues but highly expressed in cervical cancer [78,79]. In the ERα36-HMGA2 axis, HMGA2 serves as a critical downstream effector of the ERα36/EGFR/AKT pathway [8]. Estrogen signaling via ERα36 promotes HMGA2 expression, which in turn alters chromatin structure to facilitate the binding of other transcription factors to the promoters of cell cycle genes (e.g., CCNA2) [78,79]. The HMGA2 locus is a frequent site for HPV DNA integration [79]. This integration often disrupts the 3′UTR of HMGA2, removing the let-7 miRNA binding sites and leading to massive HMGA2 protein accumulation [79,80]. This synergy between estrogenic induction and viral-induced genetic stabilization drives the transition from CIN3 to invasive cancer. As a nuclear-binding oncofetal protein, HMGA2 has also been found to be related to various malignant tumors such as colorectal [81], breast [82], pulmonary [83], ovary [84] and endometrial cancer [85]. The oncogenic effect of ERα36 was attenuated after HMGA2 knockdown, which indicate that HMGA2 plays an oncogenic role in tumorigenesis and progression of cervical cancer. 

Orchestrating estrogen sensitivity and aneuploidy, WAPL (wings apart-like) is a regulator of the cohesin complex, essential for sister chromatid cohesion and DNA repair [86]. It is a protein that destabilizes cohesin binding to chromatin, but is normally inhibited during early mitosis to maintain stable sister chromatid cohesion [87,88]. Thus, WAPL is an essential factor for chromatin structure and chromosome segregation. WAPL overexpression is one of the earliest events in cervical carcinogenesis. HPV E6/E7 increases WAPL expression [89,90], and WAPL further causes chromosomal instability in the process of HPV-infected precancerous lesions to invasive cancer [91]. HPV E7 protein directly contributes to WAPL upregulation. The combined effect of WAPL-induced cohesin instability and E7-mediated centrosome amplification leads to profound chromosomal instability (CIN), a hallmark of estrogen-dependent HPV progression [86,92]. On the other hand, it has been demonstrated that WAPL increases cellular sensitivity to estrogen and its receptor by upregulating MACROD1 (LRP16) [86,92]. This sensitizes the ESR1 (ERα) pathway, allowing even physiological levels of estrogen to drive aberrant proliferation [86]. WAPL *per se* can induce CIN while HPV E6/E7 is absent. As mentioned above, WAPL increases ERα sensitivity by activating MACROD1, a co-activator of ERα. This upregulates the expression of MYC and Cyclin D1, both of which are oncogenes that regulate cell proliferation and the cell cycle [93]. Based on these facts, it can be inferred that WAPL not only induces chromosomal instability in cervical cancer tumorigenesis, but also plays a key role in activating estrogen receptor signaling in early tumorigenesis.

In summary, these mediators can be framed as a cohesive network: WAPL initiate the sensitivity to the hormonal microenvironment; ERα36 provides the rapid signaling machinery to bypass classical ERα limitations; and HMGA2 acts as the nuclear executor that translates these signals into permanent epigenetic and transcriptional changes [8,57,86].

The transition from high-risk HPV infection to invasive cervical cancer is a multistep process driven by the synergistic integration of viral oncogenesis and host hormonal signaling. HPV genome integration acts as a critical genomic “trigger,” frequently disrupting the viral E2 gene, which leads to the uncontrolled constitutive expression of the E6 and E7 oncogenes [75,76]. This integration not only facilitates host genomic rearrangements but also initiates a profound “crosstalk” with estrogen signaling [94].

While ERα expression is often lost in epithelial cancer cells, it is significantly retained and activated in the stromal tumor microenvironment (TME) [10,55]. Estrogen signaling through stromal ERα induces the secretion of paracrine factors and pro-inflammatory cytokines (e.g., IL-6, IL-8, and CXCL12), which reprogram the TME into a pro-tumorigenic niche [10,95]. This “TME remodeling” is characterized by enhanced angiogenesis, recruitment of tumor-associated macrophages (TAMs), and immune evasion, collectively providing the necessary metabolic and structural support for the survival and invasion of HPV-transformed cells [94,95]. Thus, the integration of the HPV genome provides the intrinsic oncogenic drive, while estrogen-dependent TME remodeling provides the extrinsic permissive environment for carcinogenesis [10,55].

The HPV E2 protein is a multi-functional regulatory protein that serves as the “master controller” of the papillomavirus life cycle. It is a sequence-specific DNA-binding protein (approximately 42–45 kDa) that coordinates viral DNA replication, transcriptional regulation, and genome maintenance. In the context of the estrogen-HPV crosstalk, it is important to clarify that while the HPV E2 protein is a central regulator of the viral life cycle, the most characterized physical and functional binding between the virus and the Estrogen Receptor alpha (ERα) involves the N-terminal Transactivation Domain (TAD) of the E2 protein [10,95].

Specifically, current evidence highlights the following biochemical and molecular mechanisms. The N-terminal domain (approximately the first 200 amino acids) of the HPV E2 protein is the primary region responsible for protein-protein interactions with host cellular factors [10,96]. Research indicates that this domain facilitates the recruitment of the ERα signaling complex to the viral Long Control Region (LCR). This interaction is critical because it allows estrogen to modulate the viral early promoter activity, and coordinates the E2-mediated repression (or in some stages, activation) of the E6 and E7 oncogenes in response to the hormonal environment [37,96]. Also, ERα36 can afftect stability of HPV E2 protein. While classical ERα (66 kDa) is often lost in cancerous epithelial cells, its variant ERα36 remains active. Interestingly, while E2 generally represses E6/E7, the presence of specific estrogenic signaling through ER variants can influence the turnover and stability of the E2 protein itself [8,77]. The N-terminal TAD is also the site where E2 interacts with the ubiquitin-proteasome system; thus, ER-mediated signaling can indirectly stabilize or degrade E2, thereby shifting the “brake” on viral oncogene expression. 

## 6 Estrogen-Induced Apoptosis

Apart from the pro-tumorigenic effects of E2, its anti-tumorigenic effects also draw attention, which are related to E2-induced cell apoptosis. Apoptosis is a process of eliminating unwanted cells. There are two pathways in apoptosis: intrinsic (mitochondrial) and extrinsic (death receptor) pathways. Both pathways lead to the activation of the caspase protein family, resulting in cell death [97,98]. high-dose estrogen (E2) can activate the mitochondrial pathway by binding directly to phosphodiesterase 3A (PDE3A), which stabilizes a fast-turnover protein Schlafen 12 (SLFN12). The PDE3A-SLFN12 complex binds to ribosomes, inhibiting the recruitment of signal recognition particles (SRPs), which disrupts the translation of Bcl-2 and Mcl-1. The reduction of these anti-apoptotic proteins facilitates the release of cytochrome C from mitochondria, thereby initiating apoptosis [99,100].

In addition to the post-translational mechanism via PDE3A and SLFN12, high-dose estrogen can also induce apoptosis through classical receptor-mediated signaling. As mentioned above, ERα and ERβ are the two subtypes of ER. While ERα regulates carcinogenesis, the ERβ function is thought to be antiproliferative and proapoptotic. In ovarian cancer, ERβ expression decreases during carcinogenesis. ERβ overexpression reduces proliferation and induces caspase-3–dependent apoptosis, partly through upregulation of forkhead box protein L2 (FOXL2, a suppressor oncogenic factor). High-dose estradiol triggers both genomic ERβ signaling and non-genomic GPER pathways that converge on FOXL2 activation [101]. In endometrial cancer, ERβ downregulation correlates with increased proliferation and decreased TATA-Box binding protein associated factor 9 (TAF9B) expression, a p53 coactivator essential for apoptosis-related gene transcription [102,103].

As for cervical cancer, high concentration of E2 can induce apoptosis. Huang et al. have demonstrated how β-estradiol created a high E2 environment for HeLa cells *in vitro*, and mitochondrial apoptosis initiated along with caspase cascade and altered Bax/Bcl-2 ratio [104]. These results may imply the pharmacological potential of high-dose E2 in cervical cancer. E2-triggered apoptosis involves both intrinsic and extrinsic pathways and is influenced by ER subtype balance, post-translational modifications, and E2 concentration. Further research and deeper understanding of the pathways may support the potential use of E2 or its use as a therapeutic strategy in cervical cancer treatment.

## 7 Treatment of Cervical Cancer

Cervical cancer is staged according to the 2018 International Federation of Gynecology and Obstetrics (FIGO) system, which incorporates both clinical and radiologic or pathologic assessment of lymph node status [105]. The disease progresses from stage I, where the carcinoma is confined to the cervix, to stage II (invades beyond the uterus) and stage III (carcinoma involves lower third of vagina, extends to pelvic wall, causes hydronephrosis or nonfunctioning kidney, or involves pelvic or paraaortic lymph nodes). Lastly, stage IVB, characterized by distant metastasis beyond true pelvis or involvement of mucosa of bladder or rectum. This updated staging enables more precise prognostication and treatment planning.

The National Comprehensive Cancer Network (NCCN) guidelines provide the management of cervical cancer based on disease stage [11]. For early-stage disease (IA1–IB2), surgical intervention is the mainstay. Conization or extrafascial hysterectomy with lymphadenectomy is appropriate for IA1–IA2 disease. For stage IB1 (lesions of ≤2 cm with limited stromal invasion), recent data from the SHAPE trial support the use of simple hysterectomy rather than radical hysterectomy [106]. Radical hysterectomy with pelvic lymphadenectomy is recommended for IB2 tumors (≥2 cm but <4 cm). After radical hysterectomy, the risk of disease recurrence in patients with selected risk factors can be reduced with the use of adjuvant pelvic radiotherapy [107] or adjuvant chemoradiation therapy [108]. In patients desiring fertility preservation, conization with clear margins is acceptable for patients with stage IA1 through IB1 disease. Radical trachelectomy with cerclage and lymphadenectomy may be considered for patients with tumors ≥2 cm but <4 cm [109].

For locally advanced cervical cancer (stages IB3–IVA), concurrent chemoradiation therapy is the standard of care. This includes external beam radiation therapy, weekly cisplatin, and high-dose-rate intracavitary brachytherapy. Advances in conformal and intensity-modulated radiation therapy have improved the therapeutic index by minimizing radiation dose to surrounding organs, therefore, leading to fewer side effects [110]. The EMBRACE-I trial demonstrated the efficacy and reduced morbidity of MRI-guided brachytherapy [111,112].

Immunotherapy and targeted agents are treatment options for cervical cancer. Bevacizumab, a monoclonal antibody against VEGF-A, can be used along with standard chemotherapy in recurrent or metastatic disease. Pembrolizumab, an immune checkpoint inhibitor (ICI), has demonstrated clinical benefit in both locally advanced and metastatic cervical cancer. Second-line therapies for recurrent disease include ICIs or antibody–drug conjugates such as tisotumab vedotin [113] and trastuzumab deruxtecan [114].

Overall, staging-directed, multidisciplinary treatment strategies, increasingly incorporating precision immunotherapy, have improved outcomes for patients with cervical cancer. However, many detailed cellular and molecular mechanisms underlying cervical precancer and cancer seem complicated and unexplored and warrant further investigation to develop novel therapies.

Beyond established therapies such as surgery, radiotherapy, chemotherapy, bevacizumab or pembrolizumab, the cervical cancer treatment landscape is shifting toward precision-targeted Antibody-Drug Conjugates (ADCs) and Adoptive Cell Therapies (ACTs). Tisotumab vedotin, a first-in-class ADC targeting Tissue Factor, has demonstrated superior overall survival (OS) compared to chemotherapy in the Phase 3 innovaTV 301 trial, reducing the risk of death by 30% [115,116]. Emerging ADCs also target HER2 (e.g., Trastuzumab deruxtecan) and TROP-2, providing novel options for pre-treated patients [115]. Simultaneously, cell-based strategies like Tumor-Infiltrating Lymphocyte (TIL) therapy (e.g., Lifileucel) and E7-targeted T-cell receptor (TCR)-T cells are showing promise in eradicating HPV-positive metastatic lesions by leveraging the patient’s own immune system to target viral oncoproteins [117]. Furthermore, therapeutic HPV vaccines are being refined to break immune tolerance in established malignancies [117]. These therapies represent advances from systemic cytotoxicity toward molecularly-driven and virally-targeted interventions.

## 8 Bevacizumab

Bevacizumab is a recombinant humanized monoclonal antibody that binds to vascular endothelial growth factor A (VEGF-A), thereby inhibiting its interaction with VEGF receptors on endothelial cells. 

Angiogenesis is a fundamental process in the development and progression of solid tumors [118]. During the process of oncogenesis, the area of neoplastic cells may develop hypoxia due to competition of cells for oxygen [119]. Angiogenesis is a way of coping with such a situation. There are multiple biomolecules that promote angiogenesis, including growth factors, such as VEGF, fibroblast growth factor, transforming growth factor, hepatocyte growth factor [120]. The VEGF is the most typical regulator in tumor angiogenesis [121], which can mediate vascular permeability, angiogenesis, and lymphogenesis [122]. The VEGF family has five isoforms, including VEGF-A, VEGF-B, VEGF-C, VEGF-D, and VEGF-E. They are generated by alternate splicing of a single gene. VEGF-A is the most crucial factor that maintains human endothelial function and promotes cell mitosis and vascular permeability [123]. VEGF-A mediates angiogenesis through binding to two receptor tyrosine kinases (RTKs), VEGFR-1 and VEGFR-2. The VEGF-A/VEGFR-2 axis is the primary signaling pathway regulating both physiological and pathological angiogenesis. It promotes endothelial cell proliferation, chemotaxis, and morphogenesis, and plays a pivotal role in tumor-induced angiogenesis [124]. Therefore, VEGF-A/VEGFR-2 is a popular therapeutic target in the major research of angiogenic inhibitors. 

As mentioned above, hypoxia is a prominent feature of the cervical cancer microenvironment, contributing to tumor progression and therapeutic resistance. Under hypoxic conditions, the degradation of hypoxia-inducible factor-1 alpha (HIF-1α) is inhibited, allowing it to form a heterodimer with HIF-1β. Subsequently, the heterodimer translocates into the nucleus, and activates the transcription of pro-angiogenic genes such as VEGF-A [125]. In addition, the HPV oncoproteins E6 enhance HIF-1α upregulation via the extracellular signal-regulated kinase 1/2 (ERK) signaling pathway [126]. Both E6 and E7 could interfere with the synergistic formation of the ubiquitin ligase complex that marks HIF-1α for degradation [127]. These combined pathways lead to increased VEGF-A production, which helps explain why targeting VEGF with anti-angiogenic drugs like bevacizumab can be an effective treatment strategy in cervical cancer. In the GOG 240 trial, the addition of bevacizumab to standard chemotherapy in patients with recurrent, persistent, or metastatic cervical cancer was associated with an improvement of 3.7 months in median overall survival [13]. This led to its FDA approval in 2014. 

Although Bevacizumab significantly improves survival rates, an increased risk of incidence of hypertension, thromboembolic events and genitourinary fistulas was also found. One study showed that in the population of metastatic and recurrent CC, 85.5% were classified as ineligible due to bleeding, impaired renal function, and poor performance status [128]. These limitations highlight the need for safer and more widely applicable therapeutic strategies for this challenging patient population.

## 9 Pembrolizumab

The immune checkpoint, PD-1/PD-L1 pathway is one of the most important mechanisms the body uses to maintain immune balance and prevent autoimmunity by limiting T-cell activation. In the tumor microenvironment, the cancer cells use this pathway to escape immune detection and promote tumor growth [129]. 

PD-1, a member of the immunoglobulin superfamily within the CD28/CTLA-4 family, is expressed on the surface of B-cells, T-cells, natural killer cells, dendritic cells and myeloid cells [130,131]. PD-1 exclusively interacts with its ligands PD-L1 and PD-L2. Moreover, PD-L1 is widely expressed on several kinds of cells, including hematopoietic cells [132], non-hematopoietic cells, but also tumor cells [133,134]. In contrast, PD-L2 is expressed more restricted to antigen-presenting cells like dendritic cells and macrophages. 

Upon PD-1 and PD-L1 engagement, an inhibitory signal was transmitted to attenuate T-cell receptor (TCR) signaling. The immunosuppressive effect occurs via the recruitment of Src homology region 2 domain-containing phosphatase-2 (SHP2) [135]. SHP2 is activated through the phosphorylated immunoreceptor tyrosine-based switch motifs (ITSM) and immunoreceptor tyrosine-based inhibitory motifs (ITIM) of PD-1 binding to its N-SH2 and C-SH2 domain [136]. Subsequently, activated SHP-2 inhibits TCR-mediated phosphorylation of ZAP70/CD3zeta signalosome, further attenuating TCR downstream signaling and the ability to kill tumor cells. 

Tumors take advantage of this pathway by upregulating PD-L1. Immune checkpoint inhibitors such as pembrolizumab—an anti–PD-1 humanized IgG4 monoclonal antibody—block this interaction, thereby restoring T-cell effector function and promoting tumor cell clearance. 

For locally advanced cervical cancer, on January 12, 2024, the FDA approved pembrolizumab plus chemoradiation therapy for FIGO 2014 stage III through IVA disease on the basis of findings in the KEYNOTE-A18 trial. A total of 1060 patients were randomly assigned to receive either pembrolizumab plus chemoradiotherapy (n = 529) or placebo plus chemoradiotherapy (n = 531). At a median follow-up of 29.9 months, pembrolizumab plus chemoradiation therapy was associated with significantly improved overall survival (hazard ratio for death from any cause, 0.67; 95% CI, 0.50 to 0.90; *p* = 0.004) [137].

In metastatic or recurrent cervical cancer, pembrolizumab received FDA approval on October 13, 2021. Pembrolizumab was approved to be used with chemotherapy, with or without bevacizumab, for patients with PD-L1–positive (defined as a combined positive score [CPS] ≥1) who are not candidates for curative therapy. This approval was based on data from the KEYNOTE-158 phase II trial. The study demonstrated that monotherapy with pembrolizumab has antitumor activity among 77 patients with PD-L1–positive tumors who had had disease progression after treatment for recurrent or metastatic disease (objective response, 14.3%) [138]. These findings support pembrolizumab as an important immunotherapeutic option in cervical cancer, particularly for patients with PD-L1–positive tumors and limited alternatives. Its success also emphasizes the therapeutic value of immune checkpoint blockade in cervical cancer.

Although ICIs have demonstrated clinical benefit in cervical cancer, therapeutic responses remain highly heterogeneous between patients. Estrogen signaling has been shown to promote immune suppression. This remodeling of the TME may limit the efficacy of immune checkpoint blockade, thereby contributing to variable treatment responses. Direct clinical evidence linking estrogen signaling to immunotherapy outcomes in cervical cancer is currently limited. Future studies are required to determine whether hormonal modulation can be used as a strategy to enhance immune checkpoint blockade.

## 10 Limitations

This review has several limitations. First, articles in the research literature were selected from databases by two scholars, so selection bias cannot be completely avoided. Second, the articles collected in this review are mainly in English, so the results and opinions of non-English articles may be overlooked. Finally, this study could not fully control for factors that change over time.

## 11 Future Perspective

WHO aims to eliminate cervical cancer by reducing its incidence to below 4 per 100,000 women. To achieve this goal, the 90–70–90 targets for HPV vaccination, screening, and treatment have been promoted. However, there is still a long way to go. Cervical cancer still caused nearly 350,000 deaths in 2022, with 94% occurring in low- and middle-income countries. Significant gaps between countries still persist. 

Many aspects of cervical cancer research still hold considerable potential for further advancement. The tumor microenvironment *per se* also requires further inspection. The interactions among HPV-infected keratinocytes, immune cells, and stromal cells are complex and not yet fully understood. Newer methods, such as single-cell sequencing and spatial profiling, may help define how these cell populations change during transient versus persistent HPV infection.

On the therapeutic side, combining hormonal modulation with immunotherapy may offer new opportunities. Agents such as SERMs, SERDs, or aromatase inhibitors could be explored alongside immune checkpoint inhibitors to counteract estrogen-driven immune suppression. Other approaches such as autologous T-cell therapy, therapeutic HPV vaccines, and antibody–drug conjugates with a Trop-2 inhibitor also hold promise. 

Research on cervical cancer will benefit from a comprehensive framework that incorporating HPV life cycle, hormonal signaling, immune responses, metabolism, and even the microbiome. Such research may facilitate the development of more prevention and treatment strategies.

## 12 Conclusion

Persistent infection with HR-HPV remains the fundamental driver of cervical carcinogenesis; however, viral oncogenes alone are insufficient for the transition from infection to high-grade CIN and invasive cancer. Accumulating evidence shows estrogen signaling as a crucial cofactor that amplifies HPV-induced oncogenic pathways. The interactions between estrogen and stromal cells in TME demonstrate the complex hormonal–viral crosstalk that underlies cervical cancer progression. There is a crucial need to further our knowledge on the hormonal landscape in cervical cancer. This would enhance our ability to refine preventive and therapeutic strategies, and may contribute meaningfully to ongoing efforts toward cervical cancer elimination.

## Data Availability

Not applicable.
